# Incidental findings on preoperative computed tomography for the planning of robotic arthroplasty: retrospective cohort study

**DOI:** 10.1093/bjsopen/zrag044

**Published:** 2026-09-18

**Authors:** Robin J S Jones, Jonathan R A Phillips, Matthew J Wilson, Michalis Panteli

**Affiliations:** Exeter Hip Unit, Royal Devon University Healthcare NHS Foundation Trust, Exeter, UK; Exeter Knee Reconstruction Unit, Royal Devon University Healthcare NHS Foundation Trust, Exeter, UK; Academic Department of Trauma and Orthopaedics, School of Medicine, University of Exeter, Exeter, UK; Exeter Hip Unit, Royal Devon University Healthcare NHS Foundation Trust, Exeter, UK; Exeter Hip Unit, Royal Devon University Healthcare NHS Foundation Trust, Exeter, UK; Academic Department of Trauma and Orthopaedics, School of Medicine, University of Exeter, Exeter, UK

**Keywords:** hip, knee, replacement, malignancy

## Abstract

**Background:**

The use of robotically assisted arthroplasty has increased substantially in recent years. Some robotic platforms require preoperative computed tomography for surgical planning. These scans frequently include anatomical regions beyond the primary operative field, creating potential for incidental findings. Planning computed tomography is not formally reported routinely, raising the possibility that clinically relevant incidental findings may be overlooked.

**Methods:**

The reports of all computed tomography performed for preoperative planning of robotically assisted arthroplasty at the authors’ institution between October 2019 and February 2024 were reviewed retrospectively. Incidental findings were noted, and their clinical significance assessed. The notes of patients with significant findings were reviewed to ascertain the final diagnosis and management.

**Results:**

A total of 1602 patients (mean age 66.8 years, median 68 (range 16–98) years; 847 women) were identified within the study period. Some 447 patients (27.9%) demonstrated incidental findings, 136 (8.5%) of which were significant, requiring further investigation or management. Eighty-six significant findings (5.4%) were suspicious for malignancy and, of these, five (0.3%) resulted in a new cancer diagnosis.

**Conclusion:**

In this study, the rate of significant incidental findings was 8.5% and the risk of discovery of previously undiagnosed malignancy was 0.3%. A new significant finding was demonstrated in 1 in every 12 patients, and a new cancer diagnosis in 1 in every 320 patients. Planning imaging for robotic arthroplasty should be reported by a radiologist to prevent significant findings and malignancy being missed.

## Teaser

Computed tomography (CT) for robotic arthroplasty planning frequently reveals incidental findings that are not reported routinely. Among 1602 patients, 8.5% had clinically significant incidental findings and 0.3% received a new cancer diagnosis. Routine radiological reporting of planning CT is recommended to avoid missed significant pathology.

## Introduction

The popularity of robotically assisted arthroplasty has increased exponentially in recent years, leading to a rapid expansion in the number of patients undergoing such procedures. The most commonly used platforms include MAKO^®^ (Stryker Orthopaedics, Mahwah, NJ, USA), ROSA^®^ (Zimmer Biomet, Warsaw, IN, USA), VELYS™ (DePuy Synthes, Warsaw, IN, USA), CORI™ (Smith+Nephew, Watford, UK), and OMNIBotics® (Corin Group, Cirencester, UK)^1^.

The rapid increase in the use of this technology has come with challenges. Robotically assisted arthroplasty adds surgical time, complexity, increased financial burden and, for many systems, limited surgical implant choice available to the surgeon^2,3^. Exposure to high levels of radiation for medical imaging has also been linked to carcinogenesis^4,5^. Robotically assisted surgery has been shown to improve the accuracy of implant positioning and, although there is some evidence for improved early outcomes such as less pain and soft tissue damage, the debate continues regarding cost-effectiveness and long-term outcomes^1,6–8^.

Each robotic system uses a unique surgical platform and workflow to plan and perform arthroplasty surgery. Some systems require intraoperative anatomical registration points alone, whereas others require preoperative planning imaging^1^. The MAKO^®^ robotic platform uses preoperative computed tomography (CT) combined with intraoperative registration of skeletal landmarks to enable accurate planning of implant position, bone preparation, and final implant placement^2,9–11^.

One of the unintended consequences of performing any form of medical imaging is the discovery of incidental findings. An incidental finding is defined by the American College of Radiology (ACR) as ‘an incidentally discovered mass or lesion, detected by CT, magnetic resonance imaging, or other imaging modality performed for an unrelated reason’^12^. The rate of discovery and subsequent diagnostic and economic burden of incidental findings have been demonstrated previously across multiple imaging modalities^12–16^. In whole-body trauma CT, 44.5% of patients imaged were found to have an incidental finding, with 7% of the cohort having clinically significant findings requiring further investigation or management^15^. In a 2006 study, Xiong *et al*.^16^ reported a similar rate of discovery in patients undergoing CT colonography. In that cohort, 50.5% of patients had incidental, extracolonic findings, and the cost of further investigation and management of these incidental findings was shown to double the cost of performing CT colonography^16^.

The aim of this study was to determine the rate of discovery and to assess the clinical significance of incidental findings on CT undertaken for preoperative planning of robotically assisted hip and knee arthroplasty. An understanding of the potential risk to patients from missed incidental findings is key to ensuring safe practice as the technology of hip and knee arthroplasty evolves.

## Methods

### Patient cohort

Following institutional board approval (IRB 24-1419), a retrospective study was carried out identifying all consecutive patients undergoing CT for preoperative planning of total hip arthroplasty (THA), total knee arthroplasty (TKA), and partial knee arthroplasty (PKA) between 23 October 2019 and 29 February 2024, at the Royal Devon and Exeter Hospital and Exeter Nuffield Hospital. All study methods and results were reported in accordance with STROBE guidelines for observational studies; the STROBE checklist can be found in the *Supplementary material*.

All identified studies were reviewed using Picture Archiving and Communication System software (InSight PACS, Insignia Medical Systems, ResMD, BridgeHead Software). Patient demographics and type of operation were recorded. All reports were reviewed to identify the presence of incidental findings and determine their clinical significance. Duplicate imaging for subsequent arthroplasty was excluded from further analysis but, in the case of CT performed for hip arthroplasty following knee arthroplasty imaging, the hip arthroplasty CT was used as this provided a wider field of view of the pelvis where the presence of incidental findings was more likely.

### Imaging protocols

**Fig. 1:**
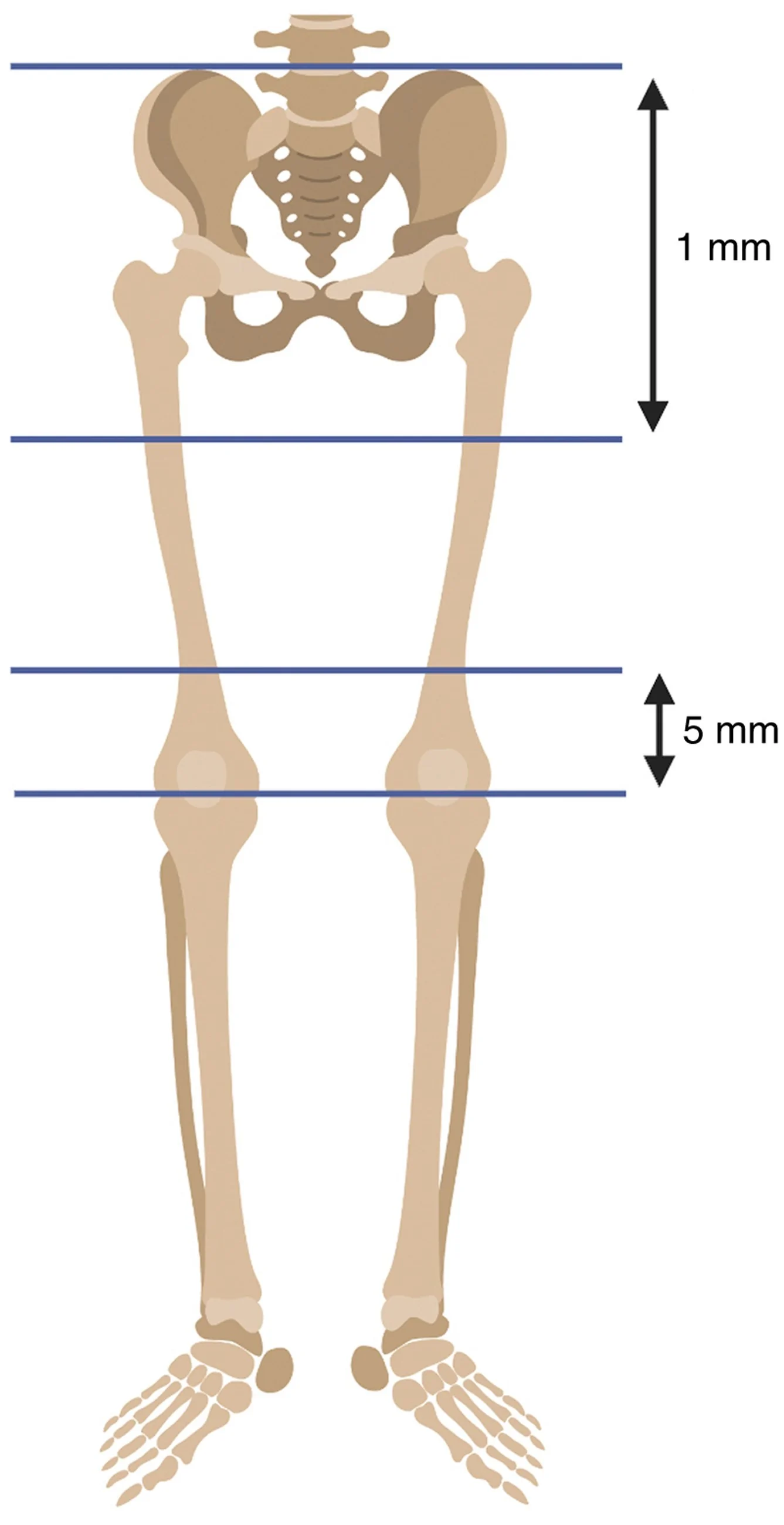
Total hip arthroplasty planning computed tomography diagram Schematic representation of the CT imaging protocol used for MAKO robotic-assisted total hip arthroplasty. The pelvic CT field of view extends from the iliac crests to at least 180 mm distal to the lesser trochanter, acquired with 1-mm slice intervals. Additional distal femoral images are obtained using 5-mm slice intervals. The diagram illustrates the anatomical regions included in the scan and the respective slice thicknesses used for pre-operative planning.

**Fig. 2:**
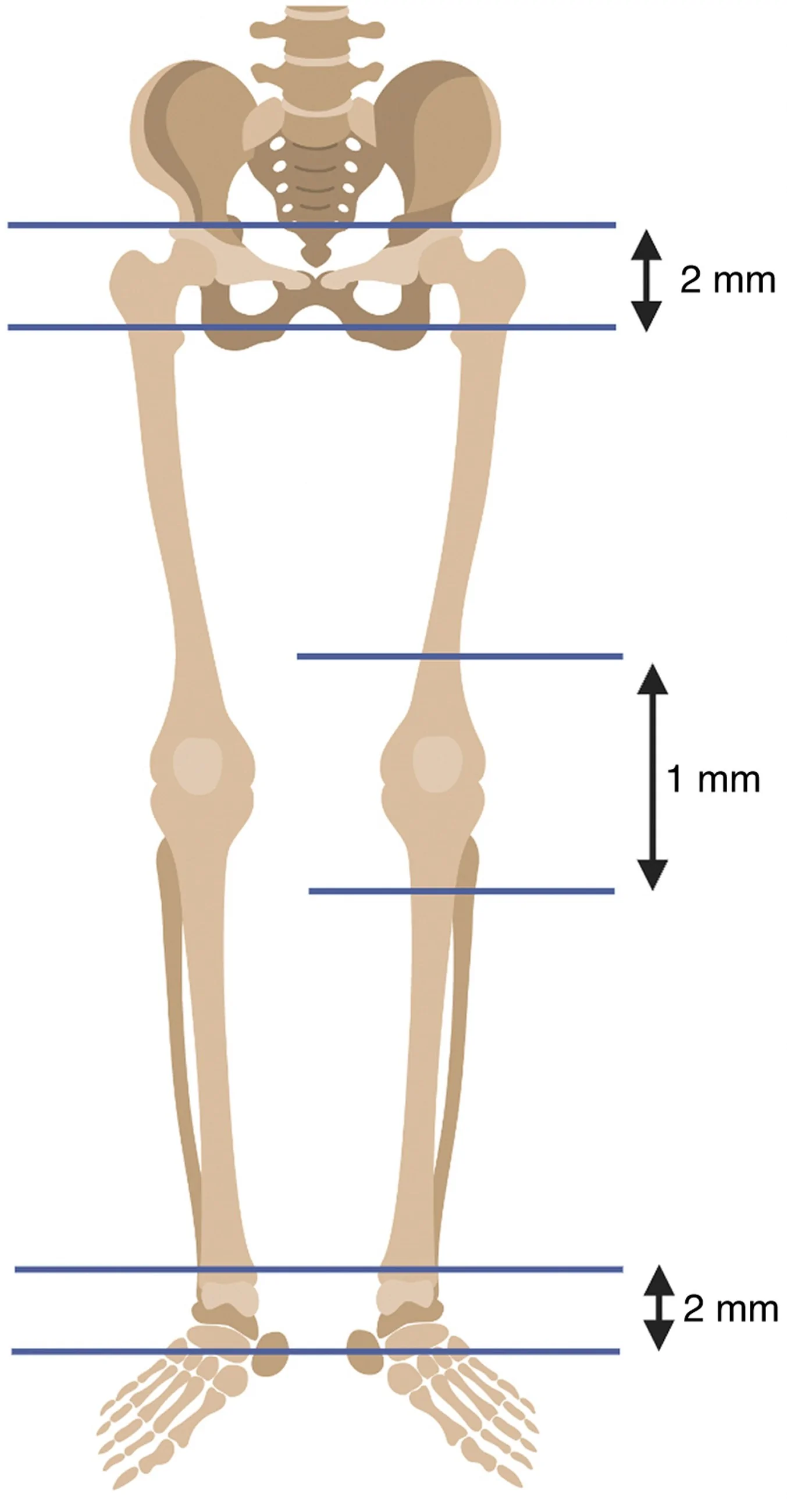
Total/partial knee arthroplasty planning computed tomography diagram Schematic representation of the CT imaging protocol used for robotic-assisted total knee arthroplasty planning. The pelvic CT field of view extends from the superior aspect of the acetabula to the proximal extent of the lesser trochanter and is acquired using 2-mm slice intervals. The operative knee is imaged separately, with the field of view extending 100 mm proximal and 100 mm distal to the joint line, acquired using 1-mm slice intervals. Both ankle joints are imaged from the level proximal to the tibial plafond to the mid-calcaneal level using 2-mm slice intervals. The diagram illustrates the anatomical regions included in the scan and the corresponding slice intervals used for pre-operative planning and alignment assessment.

All CT was done using a MAKO^®^-specific protocol as specified by the manufacturer (Stryker Orthopaedics), without contrast and with fields of view specific to the joint of interest^9,11^. During THA planning CT, the entire pelvis was imaged, with a field of view extending from the iliac crest to at least 180 mm distal to the lesser trochanter in high resolution (1-mm slice interval). Further lower-resolution images (5-mm slice interval) of the distal femur were also obtained (*Fig. 1*). Planning imaging for TKA/PKA included a smaller volume of pelvis, from the top of the acetabula to the proximal extent of the lesser trochanter (2-mm slice interval). The operative knee alone was imaged, with a field of view extending 100 mm on either side of the joint line (1-mm slice interval). Both ankle joints were imaged from proximal to the tibial plafond to the mid-calcaneal level (2-mm slice interval) (*Fig. 2*). All planning imaging was formally reported prospectively, before planned arthroplasty surgery by one of a group of five specialist, fellowship-trained, consultant musculoskeletal (MSK) radiologists.

### Classification of incidental findings

Incidental findings comprised previously unknown findings unrelated to the patient’s established medical history, as defined by the ACR^12^. Incidental findings were further classified as either significant or insignificant, with further subgrouping of significant findings as either benign or potentially representative of malignancy.

Insignificant findings were defined as those for which no further investigation or management was indicated. Examples of incidental findings deemed insignificant included fat-containing hernias, uterine fibroids, and uncomplicated diverticular disease.

Significant findings were defined as those likely to require further investigation or management. Examples of significant non-malignant findings included aneurysmal changes within the aorta and imaged vessels. Findings suspicious for malignancy included cystic changes within solid organs of the abdomen or pelvis, lytic or sclerotic bone lesions, and concerning enlargement or calcification of the prostate. Findings were deemed to be suspicious for new malignancy if cancer was within the differential diagnosis. Medical notes and subsequent investigations of all patients with significant incidental findings were reviewed to confirm the final diagnosis and further management. Any malignancy confirmed with further imaging or investigation was recorded.

### Patient follow-up

All reports with significant findings were flagged by the reporting radiologist and/or referring orthopaedic consultant. The patient was informed of the findings and a further management plan. Patients were subsequently referred to their general practitioner, or another specialist according to the type of finding, the urgency for referral, and the patient’s wishes. The need to postpone surgery was discussed with the patient and the teams involved. In the event of further investigations or need for surgery, patients were followed up by the corresponding specialist teams.

### Statistical analysis

Data collected were entered into an electronic database. Basic demographic data are presented as count with percentage or as mean(standard deviation). Count data were analysed using Pearson’s χ^2^ test, with *P* < 0.050 considered significant. Statistical analysis was undertaken using R 3.6.0 (R Foundation for Statistical Computing, Vienna, Austria).

## Results

The final study cohort comprised 1726 CT scans, performed in 1602 patients (847 (52.9%) female), with a mean age of 66.8 (median 68, range 16–98) years. Some 719 patients underwent THA, 641 had TKA, and 242 had PKA (*Table 1*).

**Table 1:** Patient demographics

	No. of patients	Sex		Age (years)	
		Male	Female	Mean(s.d.)	Median (range)
Overall	1602	755 (47.1%)	847 (52.9%)	66.8(11.2)	68 (16–98)
**Planned procedure**					
THA	719 (44.9%)	317 (44.1%)	402 (55.9%)	63.1(12.3)	64 (16–89)
TKA	641 (40.0%)	311 (48.5%)	330 (51.5%)	70.4(9.1)	71 (40–98)
PKA	242 (15.1%)	127 (52.7%)	115 (47.5%)	68.3(8.9)	69 (46–86)
**Sex**					
Male	755 (47.1%)	–	–	66.8(11.0)	68 (16–98)
Female	847 (52.9%)	–	–	66.8(11.3)	68 (21–91)

Values are *n* (%) unless otherwise stated. s.d., Standard deviation; THA, total hip arthroplasty; TKA, total knee arthroplasty; PKA, partial knee arthroplasty.

Incidental findings were discovered in 447 patients (27.9%), with 136 (8.5%) of these deemed significant, requiring further investigation or management. Eighty-six significant findings (5.4%) were suspicious for previously undiagnosed malignancy and, of these, five (0.3%) resulted in a new cancer diagnosis (*Table 2*).

**Table 2:** Incidental findings according to age, sex, procedure, and location

	Overall (*n =* 1602)	Incidental findings (*n =* 447)	Significant findings (*n =* 136)	Potential malignancy (*n =* 86)	Confirmed malignancy (*n =* 5)
**Age (years)**					
< 65	631 (39.4%)	147 (23.3%)	41 (6.5%)	27 (4.3%)	1 (0.2%)
≥ 65	971 (60.6%)	300 (30.9%)	95 (9.8%)	59 (6.1%)	4 (0.4%)
**Sex**					
Male	755 (47.1%)	247 (32.7%)	87 (11.5%)	47 (6.2%)	4 (0.5%)
Female	847 (52.9%)	200 (23.6%)	49 (5.8%)	39 (4.6%)	1 (0.1%)
**Planned procedure**					
THA	719 (44.9%)	235 (32.7%)	75 (10.4%)	44 (6.1%)	2 (0.3%)
TKA	641 (40.0%)	155 (24.2%)	46 (7.2%)	32 (5.0%)	3 (0.5%)
PKA	242 (15.1%)	57 (23.6%)	15 (6.2%)	10 (4.1%)	0 (0%)
**Location of incidental findings**					
Pelvis		401 (89.7%)	126 (92.6%)	80 (93.0%)	5 (100%)
Knee		34 (7.6%)	9 (6.6%)	5 (5.8%)	0 (0%)
Ankle		12 (2.7%)	1 (0.7%)	1 (1.2%)	0 (0%)

Values are *n* (%). THA, total hip arthroplasty; TKA, total knee arthroplasty; PKA, partial knee arthroplasty.

Of the five patients in whom a new diagnosis of cancer was made, three men were diagnosed with prostate cancer (1 of whom was also diagnosed with concurrent lymphoma); a further man was diagnosed with renal cell carcinoma and one woman was diagnosed with uterine cancer, with an additional diagnosis of ovarian cancer in the same patient following histological analysis. Two of the three patients diagnosed with prostate cancer underwent resection with curative intent, whereas the third patient was started on lifelong hormone therapy, with good treatment response. The patient with a new renal cancer had a nephrectomy with curative intent. The patient found to have uterine cancer underwent hysterectomy and bilateral salpingo-oophorectomy with curative intent, and the histology of the tissues excised demonstrated the additional presence of ovarian cancer. Nine patients were found to have significant aneurysmal changes requiring onward referral for vascular opinion. One of these underwent aneurysm repair, whereas the remaining patients were entered into surveillance.

To investigate the effect of age on the risk of incidental findings, patients aged less than 65 years were compared with older patients (*Table 3*). There were significantly more incidental findings in the group aged ≥ 65 years, both with regard to overall findings (*P* = 0.005) and significant findings (*P* = 0.028), but the difference was not significant in findings suggesting potential malignancy (*P* = 0.130) or confirmed malignancy (*P* = 0.363).

**Table 3:** Incidental findings grouped by age

Age (years)	Overall (*n =* 1602)	Incidental findings (*n =* 447)	Significant findings (*n =* 136)	Potential malignancy (*n =* 86)	Confirmed malignancy (*n =* 5)
< 65	631 (39.4%)	147 (23.3%)	41 (6.5%)	27 (4.3%)	1 (0.2%)
≥ 65	971 (60.6%)	300 (30.9%)	95 (9.8%)	59 (6.1%)	4 (0.4%)
P*		0.005	0.028	0.130	0.363

Values are *n* (%). *χ^2^ test.

Regarding the topography of the significant findings, bone lesions represented the majority of significant incidental findings, mainly in the form of lytic or sclerotic lesions, with most being investigated and found to be benign. Intrapelvic contents (bowel, prostate in men, and ovaries in women) were the next more common findings. Lesions suspicious for new malignancy were most commonly found in bone and the ovaries. Findings leading to new cancer diagnoses are listed by tissue in *Table 4*.

**Table 4:** Significant incidental findings by organ system

	No. of significant findings	Potential malignancy	Confirmed malignancy*
Bone	32	17	1
Prostate	19	9	3
Bowel	19	10	0
Ovaries	18	17	1
Soft tissue	13	12	0
Vascular	10	–	–
Uterus	8	8	1
Lymph Nodes	7	8	1
Bladder	5	2	0
Kidney	3	3	1
Testicle	2	0	0
			
Total	136	86	8

*Two patients had more than one confirmed malignancy.

Patients who underwent planning CT twice or more for multiple arthroplasties were reviewed after exclusion from the main cohort to assess for discrepancies between CT reports. Sixteen patients were identified with incidental findings noted in one CT report but not other(s). None of the discrepancies identified related to significant findings or findings suspicious for malignancy.

## Discussion

This study investigated the risk of incidental findings on preoperative CT for robotically assisted arthroplasty. Incidental findings were discovered in 27.9% of imaging performed, with significant findings that required further investigation in 8.5% of patients. Five new malignancies were identified (0.3%). These results are in keeping with previously published literature (*Table 5*)^6,17–19^. The rate of incidental findings in this study (27.9%) was lower than reported by Tran *et al*.^19^ (51.7%), Hassebrock *et al*.^6^ (45.7%), and Abdelfadeel *et al*.^17^ (34.7%), but the rate of significant findings (8.5%) was higher than documented by Qadiri *et al*.^18^ (1.6%), Tran *et al*.^19^ (2.9%), and Abdelfadeel *et al*.^17^ (0.5%). The incidence of new malignancy (0.3%) was comparable to that reported by Tran *et al*.^19^ (0.2%). Qadiri *et al*.^18^ found no new suspicious lesions, whereas Hassebrock *et al.*^6^ described the discovery of three concerning lesions, none of which represented cancer. Abdelfadeel *et al*.^17^ described a single concerning cystic lesion for which further ultrasonography was recommended; no follow-up of this patient was recorded to confirm or refute malignancy. Small sample sizes, differences in percentages receiving THA and TKA, and inconsistencies in the definitions of incidental and significant incidental findings may account for these differences.

**Table 5:** Literature review of incidental findings in planning CT for robotically assisted arthroplasty

Reference	Year	No. of patients	Age (years)*	THA	PKA/TKA	Incidental findings	Significant findings	New malignancy
Present Study	2026	1602	66.8 (68; 16–98)	719 (44.9%)	883 (55.1%)	447 (27.9%)	136 (8.5%)	5 (0.3%)
Qadiri *et al*.^18^	2023	1519	66	239 (15.7%)	1280 (84.3%)	73 (4.8%)	25 (1.6%)	0 (0%)
Tran *et al*.^19^	2021	1432	65	501 (35.0%)	931 (65.0%)	740 (51.7%)	41 (2.9%)	3 (0.2%)
Abdelfadeel *et al*.^17^	2020	176	n.a.	0 (0%)	176 (100%)	61 (34.7%)	1 (0.5%)	n.a.
Hassebrock *et al*.^6^	2020	573	65.6	78 (13.6%)	495 (86.4%)	262 (45.7%)	n.a.	0 (0%)

Values are *n* (%) unless otherwise stated; *values are mean (median; range). CT, computed tomography; THA, total hip arthroplasty; PKA, partial knee arthroplasty; TKA, total knee arthroplasty; n.a., not assessed.

Despite comparable sex representation in the present cohort, male patients were noted to have a higher incidence of incidental findings (32.7 *versus* 23.6%). Male patients were also noted to have a higher incidence of significant incidental findings (11.5 *versus* 5.8%), potentially malignant findings (6.2 *versus* 4.6%), and confirmed malignancy (0.5 *versus* 0.1%). An explanation for this disparity is likely to be the differing structures at risk imaged by planning CT: in male patients the prostate and testes are imaged by both THA and knee arthroplasty protocol CT, whereas in female patients the ovaries are only covered by the field of view of THA planning CT. This male preponderance for incidental findings is in keeping with previously published studies^17^.

Planning imaging for THA identified incidental findings at a higher rate than that undertaken for TKA, with 32.7% of studies showing incidental findings compared with 24.2%, significant findings in 10.4% for THA compared with 7.2% for TKA, and potentially malignant findings in 6.1 *versus* 5.0%. Despite the higher rate of discovery of incidental findings in imaging for THA, the rate of confirmed malignancy was higher in planning CT for TKA, with 0.5% of studies confirming new cancer diagnoses compared with 0.3% in imaging for THA. As already mentioned, the discrepancy in discovery rate of incidental findings is likely explained by the difference in field of view between THA and TKA planning CT^9,11^. The higher rate of new cancer diagnoses in the TKA cohort is likely due to the higher average age of patients undergoing knee arthroplasty compared with THA (70.4 *versus* 63.1 years), a finding previously reported in the literature^17^. TKA planning imaging revealed two new cases of prostate cancer, and a case of uterine and ovarian cancer, whereas THA planning imaging discovered a new renal cell carcinoma and a new diagnosis of metastatic prostate cancer.

Regarding topography of the lesions, most incidental findings were discovered within the pelvis (89.7%). All confirmed malignancies were also within the pelvis, an expected finding given the relatively high incidence of prostate and gynaecological malignancies^20^, compared with bone and soft tissue primary tumours likely to be found in the lower limbs^21,22^.

Previous work suggested that the rate of discovery of significant incidental findings is low and it is not cost-effective to report every planning CT formally, although this study was a relatively small case series focused solely on planning CT for total knee replacement.^18^ It was noted that the cost of reporting planning CT only represented 10% of the total cost of the scan itself, a small figure compared with both the total cost of a robotically assisted arthroplasty and the potential consequences of incidental findings being missed.

According to UK Ionising Radiation (Medical Exposure) Regulations (IRMER) guidelines, all imaging that is requested must have a ‘clinical evaluation’ of the findings documented in the patient’s medical notes^23^. This evaluation may take the form of a formal report or a documented written assessment by the referrer, and must be undertaken by a clinician ‘suitably trained and entitled’ to interpret the imaging modality in question^23^. Similar guidance has been produced by the ACR stating that clinicians reporting CT must have formal training in doing so and be fellowship-trained in a specialty that requires interpretation of such imaging, in this instance abdominal and pelvic CT^24^. If planning CT imaging is not formally reported, the referring surgeon alone is responsible for reviewing and interpreting the study and its findings, including imaging within the organs of the pelvis.

This study raises significant medicolegal concerns. If a clinically significant abnormality identified on a planning CT scan is not reported, diagnosis and subsequent treatment may be delayed. This could lead to an unfavourable prognosis or change in treatment options. It also raises the question of whether previously unreported planning CT should be reviewed retrospectively, in order to identify potentially missed lesions. The orthopaedic surgeon and their employing institution could potentially be liable for any diagnostic delay.

The discovery and subsequent investigation of incidental findings is likely to introduce patient anxiety and runs the risk of potential overinvestigation of lesions outside the referring clinician’s area of expertise. It is therefore key for robust reporting pathways and referral criteria to be implemented in departments performing robotic arthroplasty to ensure that only appropriate further investigations are organized and that delays in investigation and diagnosis are minimized.

In the UK, the best practice guidance for robotic MSK surgical service published by the British Orthopaedic Association, Royal College of Surgeons of England, and Royal College of Surgeons of Edinburgh, in respect of whether reporting of CT should be carried out states: ‘there is insufficient evidence in the literature to suggest a particular approach’^25^. There is a compelling argument that the findings of this study are reviewed by governing/regulatory bodies (such as the national associations of orthopaedic surgeons and radiologists) with the aim of updating and/or producing formal guidance on the need for reporting planning CT required for robotically assisted hip and knee arthroplasty.

This study represents the largest series of sequential patients undergoing planning CT for robotically assisted arthroplasty, and thus is more likely to provide generalizable data than previously published case series. It covers an equal mix of male and female patients, a wide range of ages, and a wide variety of planned interventions including THA, TKA, and PKA. Additionally, all scans were reported by fellowship-trained MSK radiologists, using clear definitions for the different types of incidental findings. Moreover, only the first scan/THA scan was included if patients had multiple scans for different procedures, avoiding duplicate entries. A multicentre study trial with a much larger sample size, on the other hand, would further strengthen the validity of the results.

The study has several limitations, most notably the fact that, despite all CT being reported prospectively, data review was undertaken retrospectively, potentially introducing bias. Although standardized definitions were applied, classification of incidental findings involves clinical judgement and may vary between institutions and observers. Differences in thresholds for defining significant findings and differences in interpretation of these thresholds limit direct comparison with other published series. A further limitation relates to the lack of external validity, as reporting practices may differ across countries. Moreover, the absence of internationally recognized guidelines hinders the standardization and interpretation of these reports. Furthermore, this study has not explored the potential costs involved with reporting planning CT, both in terms of increased workload for MSK radiologists and costs incurred by further investigation and follow-up required when incidental findings are discovered. Finally, although the Royal College of Radiologists (RCR) and American College of Radiology (ACR) have clear guidance on which imaging modalities require formal reports, radiological practice is likely to vary significantly between countries and healthcare systems.

## Supplementary Material

zrag044_Supplementary_Data

## Data Availability

Data are available from the corresponding author upon reasonable request.
