# Supplementary material for: Incidental findings on preoperative computed tomography for the planning of robotic arthroplasty: retrospective cohort study
Source: BJS Open. 2026 Sep 18;10(5):zrag044. doi: 10.1093/bjsopen/zrag044 (PMC13586754; doi:10.1093/bjsopen/zrag044)
Supplement: Supplementary file 1 [file zrag044_supplementary_data.docx]

**Title:** Incidental findings on pre-operative CT imaging for the planning of robotic arthroplasty: retrospective cohort study

**Authors**

Robin JS Jones^1^ (robinjones1@nhs.net), Jonathan RA Phillips^1^ (jonphillips@nhs.net), Matthew J Wilson^1^ (matthewwilson@nhs.net), Michalis Panteli^1,2^

^1^ Exeter Hip Unit, Royal Devon University Healthcare Foundation Trust, Exeter, Devon, United Kingdom ^2^Academic Department of Trauma & Orthopaedics, School of Medicine, University of Exeter, United Kingdom

**Corresponding author.**

**Mr Michalis Panteli**	1. Consultant in Hip Surgery,

**MD, FRCS (Eng), PhD**	Exeter Hip Unit, Royal Devon University Healthcare Foundation Trust,

Barrack Road, Exeter, EX2 5DW, Devon, United Kingdom

2. Honorary Lecturer, Academic Department of Trauma & Orthopaedics,

School of Medicine, University of Exeter, United Kingdom

**ORCID ID:** <https://orcid.org/0000-0002-0905-1432>

**Twitter:** @DrPanteli_Ortho

**Supplementary Materials - Index**

| **Supplementary Appendixes** |  |
| --- | --- |
| STROBE Statement | *page 2* |
|  |  |

**Supplementary Appendixes**

STROBE Statement—checklist of items that should be included in reports of observational studies

|  | Item No. | Recommendation | Page  No. | Relevant text from manuscript |
| --- | --- | --- | --- | --- |
| **Title and abstract** | 1 | (*a*) Indicate the study’s design with a commonly used term in the title or the abstract | 1 | Incidental findings on pre-operative CT imaging for the planning of robotic arthroplasty: retrospective cohort study |
|  |  | (*b*) Provide in the abstract an informative and balanced summary of what was done and what was found | 2 | The reports of all CTs performed for pre-operative planning of robotically assisted arthroplasty at our institution between October 2019 and February 2024 were retrospectively reviewed. Incidental findings were noted, and their clinical significance assessed. The notes of patients with significant findings were reviewed to ascertain their final diagnosis and management |
| Introduction | | | |  |
| Background/rationale | 2 | Explain the scientific background and rationale for the investigation being reported | 2 | The popularity of robotically assisted arthroplasty has exponentially increased in recent years, leading to a rapid expansion in the number of patients undergoing such procedures… One of the unintended consequences of performing any form of medical imaging is the discovery of incidental findings |
| Objectives | 3 | State specific objectives, including any prespecified hypotheses | 3 | The aim of this study was to determine the rate of discovery and to assess the clinical significance of incidental findings on CT imaging performed for pre-operative planning of robotically assisted hip and knee arthroplasty. An understanding of the potential risk to patients from missed incidental findings is key to ensuring safe practice as the technology of hip and knee arthroplasty evolves |
| Methods | | | |  |
| Study design | 4 | Present key elements of study design early in the paper | 5 | a retrospective study was performed identifying all consecutive patients undergoing CT imaging for pre-operative planning of Total Hip Arthroplasty (THA), Total Knee Arthroplasty (TKA) and Partial Knee Arthroplasty (PKA) between 23/10/2019 and 29/02/2024 |
| Setting | 5 | Describe the setting, locations, and relevant dates, including periods of recruitment, exposure, follow-up, and data collection | 5 | all consecutive patients undergoing CT imaging for pre-operative planning of Total Hip Arthroplasty (THA), Total Knee Arthroplasty (TKA) and Partial Knee Arthroplasty (PKA) between 23/10/2019 and 29/02/2024, at the Royal Devon and Exeter Hospital and Exeter Nuffield Hospital. All identified studies were reviewed using Picture Archiving and Communication System software (InSight PACS, Insignia Medical Systems, ResMD, BridgeHead Software Limited). Patient demographics and type of operation were recorded. All reports were reviewed to assess the presence of incidental findings and determine their clinical significance |
| Participants | 6 | (*a*) *Cohort study*—Give the eligibility criteria, and the sources and methods of selection of participants. Describe methods of follow-up | 5 | all consecutive patients undergoing CT imaging for pre-operative planning of Total Hip Arthroplasty (THA), Total Knee Arthroplasty (TKA) and Partial Knee Arthroplasty (PKA)… All reports were reviewed to assess the presence of incidental findings and determine their clinical significance |
|  |  | (*b*) *Cohort study*—For matched studies, give matching criteria and number of exposed and unexposed | N/A | N/A |
| Variables | 7 | Clearly define all outcomes, exposures, predictors, potential confounders, and effect modifiers. Give diagnostic criteria, if applicable | 6 | Incidental findings were defined as previously unknown findings unrelated to the patient’s established medical history, as defined by the ACR (12, 13). Incidental findings were further classified as either significant or insignificant, with further subgrouping of significant findings as either benign or potentially representative of malignancy |
| Data sources/ measurement | 8* | For each variable of interest, give sources of data and details of methods of assessment (measurement). Describe comparability of assessment methods if there is more than one group | 5 | All identified studies were reviewed using Picture Archiving and Communication System software… All reports were reviewed to assess the presence of incidental findings and determine their clinical significance |
| Bias | 9 | Describe any efforts to address potential sources of bias | 6 | Incidental findings were defined as previously unknown findings unrelated to the patient’s established medical history… Insignificant findings were defined as those where no further investigation or management was indicated… Significant findings were defined as those likely to require further investigation or management… Findings were deemed to be suspicious for new malignancy if cancer was within the differential diagnosis |
| Study size | 10 | Explain how the study size was arrived at | 4 | all consecutive patients undergoing CT imaging for pre-operative planning of Total Hip Arthroplasty (THA), Total Knee Arthroplasty (TKA) and Partial Knee Arthroplasty (PKA) between 23/10/2019 and 29/02/2024, |

Continued on next page

| Quantitative variables | 11 | Explain how quantitative variables were handled in the analyses. If applicable, describe which groupings were chosen and why | 7 | Data collected was entered into an electronic database. Statistical analysis was undertaken using the computing environment R |
| --- | --- | --- | --- | --- |
| Statistical methods | 12 | (*a*) Describe all statistical methods, including those used to control for confounding | 7 | Basic demographic data were presented as count (percentage) or as mean ± SD. Count data were analysed using a Pearson’s chi square test, with a p-value of <0.05 considered significant |
|  |  | (*b*) Describe any methods used to examine subgroups and interactions | 8 | To investigate the effect of age in the risk of incidental findings, we compared patients younger than 65 years old with those aged 65 or older |
|  |  | (*c*) Explain how missing data were addressed | N/A | N/A |
|  |  | (*d*) *Cohort study*—If applicable, explain how loss to follow-up was addressed | N/A | N/A |
|  |  | (*e*) Describe any sensitivity analyses | N/A | N/A |
| Results | | | | |
| Participants | 13* | (a) Report numbers of individuals at each stage of study—eg numbers potentially eligible, examined for eligibility, confirmed eligible, included in the study, completing follow-up, and analysed | 8 | A total of 1,602 patients were included in the study (847 (52.9%) female), with a mean age of 66.8 years (median 68 years; range: 16-98 years), 719 patients undergoing THA, 641 patients undergoing TKA and 242 patients undergoing PKA |
|  |  | (b) Give reasons for non-participation at each stage | N/A | N/A |
|  |  | (c) Consider use of a flow diagram | N/A | N/A |
| Descriptive data | 14* | (a) Give characteristics of study participants (eg demographic, clinical, social) and information on exposures and potential confounders | 8 | A total of 1,602 patients were included in the study (847 (52.9%) female), with a mean age of 66.8 years (median 68 years; range: 16-98 years), 719 patients undergoing THA, 641 patients undergoing TKA and 242 patients undergoing PKA |
|  |  | (b) Indicate number of participants with missing data for each variable of interest | N/A | N/A |
|  |  | (c) *Cohort study*—Summarise follow-up time (eg, average and total amount) | N/A | N/A |
| Outcome data | 15* | *Cohort study*—Report numbers of outcome events or summary measures over time | 8 | Incidental findings were discovered in 447 patients (27.9%), with 136 (8.5%) of these deemed significant |
|  |  | *Case-control study—*Report numbers in each exposure category, or summary measures of exposure | N/A | N/A |
|  |  | *Cross-sectional study—*Report numbers of outcome events or summary measures | N/A | N/A |
| Main results | 16 | (*a*) Give unadjusted estimates and, if applicable, confounder-adjusted estimates and their precision (eg, 95% confidence interval). Make clear which confounders were adjusted for and why they were included | 8 | Incidental findings were discovered in 447 patients (27.9%), with 136 (8.5%) of these deemed significant, requiring further investigation or management. Eighty-six (5.4%) significant findings were suspicious for previously undiagnosed malignancy and, of these, five (0.3%) resulted in a new cancer diagnosis |
|  |  | (*b*) Report category boundaries when continuous variables were categorized | N/A | N/A |
|  |  | (*c*) If relevant, consider translating estimates of relative risk into absolute risk for a meaningful time period | N/A | N/A |

Continued on next page

| Other analyses | 17 | Report other analyses done—eg analyses of subgroups and interactions, and sensitivity analyses | 9 | Regarding the topography of the significant findings, bone lesions represented the majority of significant incidental findings, mainly in the form of lytic or sclerotic lesions, with most being investigated and found to be benign. Intra-pelvic contents (bowel, prostate in men and ovaries in women) were the next more common findings. Lesions suspicious for new malignancy were most commonly found in bone and the ovaries. Findings leading to new cancer diagnoses are listed by tissue in Table 4…  Patients who underwent two or more planning CTs for multiple arthroplasties were reviewed after exclusion from the main cohort to assess for discrepancies between their CT reports. Sixteen patients were identified with incidental findings reported on one CT but not other(s). None of the discrepancies noted related to significant findings or findings suspicious for malignancy |
| --- | --- | --- | --- | --- |
| Discussion | | | | |
| Key results | 18 | Summarise key results with reference to study objectives | 10 | Incidental findings were discovered in 27.9% of imaging performed, with significant findings that requiring further investigation in 8.5% of patients. Five new malignancies were identified (0.3%) |
| Limitations | 19 | Discuss limitations of the study, taking into account sources of potential bias or imprecision. Discuss both direction and magnitude of any potential bias | 13 | The study has several limitations, most notably the fact that despite all CT imaging being reported prospectively, study design and data review was performed retrospectively, potentially introducing bias. Although standardised definitions were applied, classification of incidental findings involves clinical judgement and may vary between institutions and observers. Differences in thresholds for defining ‘significant’ findings and differences in interpretation of these thresholds limit direct comparison with other published series. This study also does not explore the potential costs involved with reporting planning CTs, both in terms of increased workload for MSK radiologists and in terms of costs incurred through further investigation and follow-up required when incidental findings are discovered. Finally, whilst the RCR and ACR have clear guidance on which imaging modalities require formal reports, radiological practice is likely to vary significantly between countries and healthcare systems |
| Interpretation | 20 | Give a cautious overall interpretation of results considering objectives, limitations, multiplicity of analyses, results from similar studies, and other relevant evidence | 10 | This study investigates the risk of incidental findings on pre-operative CT imaging for robotically assisted arthroplasty. Incidental findings were discovered in 27.9% of imaging performed, with significant findings that requiring further investigation in 8.5% of patients. Five new malignancies were identified (0.3%). These results are in keeping with previously published literature (Table 5) (6, 18-20). The rate of incidental findings in this study (27.9%), was lower than Tran et al. (51.7%) (20), Hassebrock et al. (45.7%) (6) and Abdelfadeel et al. (34.7%) (18), but the rate of significant findings (8.5%) was higher (Qadiri et al. (1.6%) (19); Tran et al. (2.9%) (20); Abdelfadeel et al. (0.5%) (18)). The incidence of new malignancy (0.3%) was comparable to that reported by Tran et al. (02%) (20). The study by Qadiri et al. found no new suspicious lesions (19), whilst Hassebrock described the discovery of three concerning lesions, none of which represented cancer (6). Abdelfadeel et al. described a single concerning cystic lesion in their cohort for which a further ultrasound was recommended; no follow-up of this patient is recorded to confirm or refute malignancy (18). Small sample sizes, differences in percentages receiving THA and TKA, and inconsistencies in the definitions of incidental and significant incidental findings may account for these differences |
| Generalisability | 21 | Discuss the generalisability (external validity) of the study results | 13 | Differences in thresholds for defining ‘significant’ findings and differences in interpretation of these thresholds limit direct comparison with other published series… whilst the RCR and ACR have clear guidance on which imaging modalities require formal reports, radiological practice is likely to vary significantly between countries and healthcare systems |
| Other information | |  | | |
| Funding | 22 | Give the source of funding and the role of the funders for the present study and, if applicable, for the original study on which the present article is based | N/A | No funding source |

*Give information separately for cases and controls in case-control studies and, if applicable, for exposed and unexposed groups in cohort and cross-sectional studies.

**Note:** An Explanation and Elaboration article discusses each checklist item and gives methodological background and published examples of transparent reporting. The STROBE checklist is best used in conjunction with this article (freely available on the Web sites of PLoS Medicine at http://www.plosmedicine.org/, Annals of Internal Medicine at http://www.annals.org/, and Epidemiology at http://www.epidem.com/). Information on the STROBE Initiative is available at www.strobe-statement.org.
